# Oscillometric measurement of the ankle-brachial index and the estimated carotid-femoral pulse wave velocity improves the sensitivity of an automated device in screening peripheral artery disease

**DOI:** 10.3389/fcvm.2023.1275856

**Published:** 2023-12-12

**Authors:** Krisztina Fendrik, Katalin Biró, Dóra Endrei, Katalin Koltai, Barbara Sándor, Kálmán Tóth, Gábor Késmárky

**Affiliations:** ^1^ Division of Angiology, 1st Department of Medicine of the Clinical Centre University of Pécs, University of Pécs Medical School, Pécs, Hungary; ^2^ Division of Cardiology, 1st Department of Medicine of the Clinical Centre University of Pécs, University of Pécs Medical School, Pécs, Hungary

**Keywords:** peripheral artery disease, PAD, screening, oscillometric, BOSO

## Abstract

**Background and aims:**

To overcome the time and personnel constraints of the Doppler method, automated, four-limb blood pressure monitors were recently developed. Their additional functions, such as measuring the estimated carotid-femoral pulse wave velocity (ecfPWV), have been, thus far, less studied. We aimed to compare the sensitivity and specificity of different ankle-brachial index (ABI), toe-brachial index (TBI), and ecfPWV measurement methodologies to evaluate their contribution to peripheral artery disease (PAD) screening.

**Methods:**

Among 230 patients (mean age 64 ± 14 years), ABI measurements were performed using a Doppler device and a manual sphygmomanometer. The Doppler ABI was calculated by taking the higher, while the modified Doppler ABI by taking the lower systolic blood pressure of the two ankle arteries as the numerator, and the higher systolic blood pressure of both brachial arteries as the denominator. The automated ABI measurement was carried out using an automatic BOSO ABI-system 100 PWV device, which also measured ecfPWV. TBI was obtained using a laser Doppler fluxmeter (Periflux 5000) and a photoplethysmographic device (SysToe). To assess atherosclerotic and definitive PAD lesions, vascular imaging techniques were used, including ultrasound in 160, digital subtraction angiography in 66, and CT angiography in four cases.

**Results:**

ROC analysis exhibited a sensitivity/specificity of 70.6%/98.1% for the Doppler ABI (area under the curve, AUC = 0.873), 84.0%/94.4% for the modified Doppler ABI (AUC = 0.923), and 61.5%/97.8% for the BOSO ABI (AUC = 0.882) at a cutoff of 0.9. Raising the cutoff to 1.0 increased the sensitivity of BOSO to 80.7%, with the specificity decreasing to 79.1%. The ecfPWV measurement (AUC = 0.896) demonstrated a 63.2%/100% sensitivity/specificity in predicting atherosclerotic lesions at a cutoff of 10 m/s. Combining BOSO ABI and ecfPWV measurements recognized 89.5% of all PAD limbs.

**Conclusion:**

The combined BOSO ABI and ecfPWV measurements may help select patients requiring further non-invasive diagnostic evaluation for PAD. The user-friendly feasibility may make it suitable for screening large populations.

## 1. Introduction

Peripheral artery disease (PAD) is a progressive atherosclerotic disorder of the abdominal aorta and the arteries of the extremities, affecting the lower limbs more commonly. It is the third leading cause of atherosclerotic mortality, following coronary heart disease and stroke (1). PAD is a disease associated with a high prevalence, affecting more than 230 million individuals worldwide. In 2015, the global prevalence of PAD was estimated at 5.56% (3.79%–8.55%) (2), clearly increasing with advancing age; it can even reach 20% over the age of 80 (3). Despite its high prevalence, PAD is unfortunately often recognized late, in the stage of critical limb ischemia. The underdiagnosis can be traced back to the fact that exercise-induced intermittent claudication, which is considered a typical symptom of the disease, only occurs in about 10%–20% of the patients (4). On the other hand, it can be due in large part to the low use of conventional diagnostic tools. The accepted non-invasive gold standard to diagnose PAD is the Doppler-based ankle-brachial index (ABI) measurement (4–7). The method is cost-effective and widely available, even in primary care. However, its wide use is limited due to time and personnel constraints since correct implementation requires expertise.

ABI is defined as a ratio of the higher systolic blood pressure of the posterior tibial artery (PTA) or the dorsal pedal artery (DPA) of each lower limb and the higher systolic blood pressure of the upper limbs (4, 6, 7). However, some studies suggested that to increase sensitivity and to achieve a more accurate risk classification, the use of the modified ABI, i.e., taking the lower systolic blood pressure of the two ankle arteries as the numerator, is more appropriate (8–10).

To overcome the limitations of the Doppler method, automated, four-limb blood pressure monitors were recently developed, which are specially designed for the ABI measurement. Simultaneous blood pressure measurement on all four extremities significantly reduces the examination time (11). However, due to pressure overestimation (12) and reduced reliability in low ABI ranges (13–16), current guidelines recommend the traditional Doppler method over automated ABI measurement for PAD diagnostics (4, 6, 7).

To improve the sensitivity in detecting PAD, some automated devices have been equipped with additional functions, such as measuring the pulse wave velocity (PWV) or toe-brachial index (TBI). Thus far, a very limited amount of data has come to light in this regard.

The measurement of aortic inelasticity, in particular, the aortic PWV (PWVao), may be suitable for detecting individuals at high cardiovascular (CV) risk (17) and is considered an independent predictor of subsequent CV events (18). The non-invasive gold standard for measuring the PWVao is the measurement of the carotid-femoral pulse wave velocity (cfPWV) (19). In contrast to applanation tonometry, the traditional measurement method of the cfPWV, utilizing automated oscillometric devices requires lower operator skills and a shorter examination duration (20). According to the current guidelines of the European Society of Hypertension (ESH), a PWVao above 10 m/s can be considered an independent predictor of organ damage (21). However, PWV is not included in the current PAD guidelines (4, 6, 7).

The purpose of the present study was to evaluate the measurement accuracy, sensitivity, and specificity of an automated oscillometric device (BOSO ABI-system 100 PWV) compared to the standard Doppler method (also including the modified ABI calculation) and toe pressure measurement, taking results of vascular imaging techniques as a reference. We also aimed to investigate whether the additional estimated cfPWV (ecfPWV) function could enhance the sensitivity in PAD screening.

## 2. Materials and methods

### 2.1. Study design

A total of 230 adult patients (≥18 years) were enrolled in our study. Patients were recruited from January 2022 to November 2022 in the outpatient clinic and in the ward of the Division of Angiology at the University of Pécs Clinical Centre. The study subjects ranged in age from 23 to 92 years. Patients who did not provide written informed consent were excluded. The included individuals were divided into the following subgroups: control group, patients with previously confirmed PAD, patients with high CV risk, patients with very high CV risk, and patients with other non-atherosclerotic CV diseases. Patients of the latter three groups were not previously diagnosed with PAD. The control group consisted of non-smoking individuals matched for sex and age (within a ±5 year tolerance) who did not have diabetes and any CV diseases, except for essential, uncomplicated, medically properly treated arterial hypertension. Patients with at least moderate stenosis of the arteries of the lower extremities (luminal stenosis greater than 50% of the lumen) were considered as PAD patients (22). A history of percutaneous transluminal or surgical revascularization or amputation was not an exclusion criterion. High and very high CV risk was defined according to the 2021 European Society of Cardiology (ESC), “Guidelines on cardiovascular disease prevention in clinical practice” (23). The group of patients with non-atherosclerotic CV diseases (“other CV”) involved mainly patients treated in our angiology ward afflicted with venous thromboembolic diseases. We aimed to separate these patients, who had a low cardiovascular risk due to their young age, from the patients recruited for the control group.

### 2.2. Ethical approval and consent to participate

The investigation followed the principles of the Declaration of Helsinki and was approved by the Regional Committee for the Research Ethics of the University of Pécs (No. 9343-PTE 2022). Informed consent was obtained from all subjects prior to being included in the study.

### 2.3. Methods

Anamnestic data, concomitant medication, risk factors, and co-morbidities were assessed from every patient.

Prior to performing any instrumental investigation, subjects were acclimatized to a controlled room temperature (22–24°C) for at least 5 min in a lying position. The measurements were performed by the same experienced operator with the patients in the supine position, following the same examination sequence for all subjects.

#### 2.3.1. ABI measurements

##### 2.3.1.1. Hand-held Doppler method

Systolic blood pressure in the PTA and DPA of both legs as well as in the brachial artery of both arms was measured using a hand-held Doppler ultrasound device (Bidop ES-100V3, Hadeco Inc., Japan) operated with an 8-MHz probe and a manual sphygmomanometer following the same measurement sequence (right arm–right leg–left leg–left arm). ABI was calculated in two different ways—taking the higher or the lower systolic blood pressure in the PTA and the DPA of each ankle as the numerator, giving the Doppler ABI or the modified Doppler ABI, respectively. The higher systolic blood pressure of both arms was taken as the denominator.

##### 2.3.1.2. Automated measurement

The measurements were performed using an automated, oscillometric blood pressure monitor (BOSO ABI-system 100 PWV, Bosch & Sohn GmbH, Germany) as described in the user instructions provided by the manufacturer^1^. The blood pressure cuffs were attached to both upper arms and both ankles according to the color coding. The lower edges of the arm cuffs were positioned approximately 2–3 cm above the elbow joint, and the ones of ankle cuffs were approximately 1 cm above the ankle joints. The white marking and the tube were positioned over the PTA. During the measurement, patients were encouraged to remain silent and keep all four limbs still. The measurements of the arterial blood pressure were performed on all four extremities simultaneously based on the oscillometric principle (24).

An ABI value ≤0.9 was considered abnormal, and a value >1.4 was considered indicative of mediasclerosis.

#### 2.3.2. ecfPWV measurement with the automated oscillometric device

Upon completion of measuring the ABI in both legs, the device performed the oscillometric measurement of the ecfPWV. Through simultaneous inflation of the upper and lower cuffs, the device determined the pulse transit time between the brachial and tibial arteries by analyzing the oscillometric amplitudes (25). The pulse transit time is used to calculate the brachial-ankle pulse wave velocity (baPWV), from which the cfPWV can be estimated based on the following formula: ecfPWV = 0.833 × baPWV −2.33 (m/s) (26).

Values above a cutoff level of 10 m/s were considered abnormal (21). The user manual of the manufacturer also supported this cutoff level.

#### 2.3.3. TBI measurements

##### 2.3.3.1. Laser Doppler method

Systolic toe pressure was first measured by laser Doppler (LD) flowmetry using the linear deflation method (27) (PeriFlux System 5000, Perimed, Stockholm, Sweden) according to the manufacturer's user manuals; the analysis was performed using dedicated software (PeriSoft v2.50). The probe was positioned on the plantar surface of the distal phalanx of the first toe with the aid of a double-sided adhesive. A suitable-sized toe cuff was placed under the LD probe. After reaching a suprasystolic blood pressure of 200 mmHg, the pressure began to automatically decrease in small steps. The pressure value measured at the reappearance of the perfusion curve corresponded to the systolic toe pressure.

##### 2.3.3.2. Photoplethysmographic method

Systolic toe pressure was also measured using a portable, battery-operated device (SysToe, Atys Medical, France) by implementing the infrared photoplethysmographic (PPG) principle. The occlusion cuff was positioned on the proximal portion of the first toe, and a cuff equipped with a sensor was placed above it. Following inflation to a pressure of 300 mmHg, the occlusion cuff was automatically deflated at a specified speed. The return of blood flow was detected by the sensor in the form of a steep upward curve. The pressure measured at this point represented the systolic toe pressure^2^.

The TBI for each lower limb was determined by dividing the systolic toe pressure with the higher systolic arm pressure. A TBI value ≤0.7 was considered abnormal.

#### 2.3.4. Vascular imaging techniques

Except for preknown, chronic, non-intervenable PAD cases [19 patients, documented by a previous digital subtraction angiography (DSA)], all other patients were examined by a vascular imaging technique. 47 patients with, at minimum, Fontaine stage IIb, underwent DSA with subsequent intervention. In 160 cases, a color-coded duplex ultrasound was performed, and in four cases, a CT angiography was performed. A stenosis characterized in at least 50% was considered significant.

##### 2.3.4.1. Color-coded duplex ultrasound

A color-coded duplex ultrasound of the lower extremity arteries was performed using a GE Vivid S60N v202CH (SN 003732S60) device operated with a 10-MHz linear probe by a single investigator. Two-dimensional B-mode images and pulsed Doppler spectral waveforms with the analysis of the peak systolic velocity (PSV) were evaluated from the distal common femoral and the proximal and middle thirds of the superficial femoral artery, popliteal artery, peroneal artery, distal PTA, and DPA. Stenoses were evaluated with the PSV ratio (ratio of PSV at stenosis to the PSV measured directly proximal to the stenosis), considered as significant when >2 (28). Due to the limited appraisability of duplex ultrasound upon the calf, the Hodgkiss–Harlow classification (29) was also used to evaluate a stenosis value exceeding 50%. Atherosclerotic plaques were defined as an intima-media thickness exceeding one of the neighboring sites by at least 50% (30).

##### 2.3.4.2. CT angiography

CT angiography was performed in the Department of Medical Imaging at the University of Pécs Clinical Centre using the GE Medical Systems Revolution CT (SN REVVX2000052CN) and Siemens Somatom Perspective (SN 77934) devices, following intravenous administration of contrast agent. The multidimensional reconstruction of the abdominal, pelvic, and lower limb arteries was evaluated by experienced radiologists.

##### 2.3.4.3. Digital subtractional angiography

DSA was performed in the Department of Medical Imaging at the University of Pécs Clinical Centre using the Siemens Axiom Artis dTA (SN 55371) device. Following insertion of the catheter via a transfemoral or transbrachial approach, a contrast agent was administered intra-arterially. The obtained images of anteroposterior sequential views of the lower abdomen, pelvis, and lower extremities were evaluated by experienced angiographers. The visual stenosis estimate was performed by comparison to the normal surrounding arterial segments.

#### 2.3.5. Statistical analysis

Statistical analysis was performed using Statistical Product and Service Solutions (SPSS) statistical software, version 28.0.0.0 (SPSS Inc., Chicago, IL, USA). Continuous variables were expressed as the mean ± standard deviation (SD). Pearson's chi-squared test was used to compare the categorical variables between the subgroups. The between- and within-group analyses of continuous variables were performed by one-way ANOVA. Homogeneity of variances was analyzed by Levene's test; in cases of equal variances, Tukey’s *post-hoc* test was performed, and in cases of inhomogeneity of variances, Welch's statistics and Tamhane's *post-hoc* test were performed. The association between the Doppler-assisted and oscillometric BOSO ABI measurements was determined by the Pearson product–moment correlation, in which a correlation coefficient (*r*) greater than 0.5 was considered to demonstrate a strong correlation.

The intermodality agreement of the Doppler and BOSO ABI readings was analyzed by the Bland–Altman method (31). The proportional bias was evaluated by linear regression analysis of the differences between the two measurements.

The diagnostic efficiency of the various methods was compared using receiver operating characteristic (ROC) curve analysis. The accuracy of the diagnostic tests was estimated by the area under the curve (AUC) value. The optimal cutoff value for each method was calculated using Youden's *J* statistic based on the “sensitivity + specificity − 1” equation. The cutoff value belonging to the highest Youden's *J* index was selected. The corresponding AUC values of the independent ROC curves were compared using the Hanley–McNeil algorithm.^3^

A *p*-value <0.05 was considered to indicate statistical significance.

## 3. Results

The demographics and baseline characteristics of the study population are summarized in Supplementary Table 1. The composition of the subgroups depicts a cross section of consecutively screened patients; therefore, we did not aim to achieve homogeneity between subgroups. BMI was the highest in the high CV risk group, which significantly differed from the control (*p* = 0.005) and confirmed PAD (*p* = 0.006) groups. The proportion of current smokers did not differ between subgroups, but that of former smokers did, which was the highest in the confirmed PAD group. Some patients already had severe PAD symptoms before diagnosis. Among patients with confirmed PAD, 37 (49.3%) had previously been diagnosed with atherosclerotic disease of another vascular bed. Twelve patients (16.0%) underwent lower extremity revascularization within the 6 months preceding their inclusion in our study.

A total of 455 lower limbs of 230 patients were analyzed. ABI was not measurable in four limbs due to major amputations and in one limb due to ankle ulcerations. TBI could not be determined in 11 cases because of major/minor amputations or toe gangrene and in additional 34 cases by PPG due to technical problems.

### 3.1. Agreement of the various methods

One-way ANOVA revealed a statistically significant difference between ABI values measured by the Doppler, modified Doppler, and oscillometric methods (*p* < 0.001). The mean Doppler ABI differed significantly from the mean modified Doppler ABI and the mean BOSO ABI (Tukey’s *post-hoc* test, *p* < 0.001 for the former, *p* = 0.004 for the latter). A significant difference between BOSO and modified Doppler ABI was also found (*p* = 0.04).

TBI measured by the LD and PPG methods did not differ significantly (one-way ANOVA, *p* = 0.506).

The ABI, TBI, and ecfPWV values of patient groups are summarized in Table 1.

**Table 1:** Doppler, modified Doppler, and BOSO ABI, LD and PPG TBI, and ecfPWV values in the various subgroups of patients.

	All patients (*n* = 230)	Control (*n* = 23)	Other CV (*n* = 21)	High CV risk (*n* = 46)	Very high CV risk (*n* = 65)	Confirmed PAD (*n* = 75)
Doppler ABI	0.977 ± 0.349	1.141 ± 0.093	1.094 ± 0.109	1.069 ± 0.251	1.039 ± 0.378	0.751 ± 0.405
Modified Doppler ABI	0.846 ± 0.353	1.087 ± 0.0839	1.035 ± 0.109	0.976 ± 0.223	0.922 ± 0.336	0.544 ± 0.366
BOSO ABI	0.902 ± 0.351	1.127 ± 0.061	1.082 ± 0.105	1.024 ± 0.239	0.950 ± 0.280	0.661 ± 0.426
LD TBI	0.606 ± 0.25	0.819 ± 0.068	0.780 ± 0.172	0.734 ± 0.201	0.617 ± 0.204	0.389 ± 0.206
PPG TBI	0.618 ± 0.249	0.842 ± 0.078	0.778 ± 0.157	0.739 ± 0.202	0.626 ± 0.210	0.404 ± 0.213
ecfPWV (m/s)	10.09 ± 2.65	8.18 ± 1.17	8.31 ± 1.67	9.63 ± 2.58	11.18 ± 2.62	10.97 ± 2.63
Not measurable (*n*)	46 (20.0%)	0 (0%)	1 (4.8%)	3 (6.5%)	7 (10.8%)	35 (46.7%)

ABI, ankle-brachial index; LD, laser Doppler; PPG, photoplethysmography; TBI, toe-brachial index; ecfPWV, estimated carotid-femoral pulse wave velocity; CV, cardiovascular; PAD, peripheral artery disease.

Welch's ANOVA test showed significant differences (*p* < 0.001) for all parameters (Doppler ABI, modified Doppler ABI, BOSO ABI, LD and PPG TBI, ecfPWV) among the various patient subgroups. Tamhane's *post-hoc* testing revealed that the BOSO ABI of the confirmed PAD patients significantly differed from the values of all other subgroups (*p* < 0.001 for all comparisons). Significant differences were found upon comparing the BOSO ABI values of the control group to the high CV risk, very high CV risk, and confirmed PAD patients (*p* < 0.002 for all comparisons).

The ecfPWV of the control patients differed significantly from those of the high CV risk, very high CV risk, and confirmed PAD groups (*p* = 0.025 for the control vs. very high CV risk, *p* < 0.001 for the other comparisons). The values of the confirmed PAD patients differed significantly from the control and other CV group (*p* < 0.001 for both), but no significant difference was found between the confirmed PAD vs. high or very high CV risk patients (*p* = 0.197 for the former, *p* = 1.000 for the latter). A probable explanation is that ecfPWV was immeasurably low in 6.5% of the high CV risk, in 10.8% of the very high CV risk, and in 46.7% of the confirmed PAD patients. A significant difference was found between the high and very high CV risk patients (*p* = 0.038).

To evaluate the correlation between the Doppler, modified Doppler, and the oscillometric ABI measurements, 45 cases (9.9%) needed to be excluded, in which the BOSO ABI was “0,” while the Doppler ABI was a non-zero value. Of these cases, 28 patients were diabetic and 17 were non-diabetic (16.4% of all ABI measurements of diabetic and 6.0% of non-diabetic patients). Although these oscillometric measurements had to be classified as technically invalid, significant PAD lesions were detected in 100% of these cases by vascular imaging. A significant correlation was found between the Doppler and BOSO ABI values (*r* = 0.614, *p* < 0.001) and a slightly more pronounced correlation between the modified Doppler and BOSO ABI values (*r* = 0.641, *p* < 0.001).

**Figure 1:**
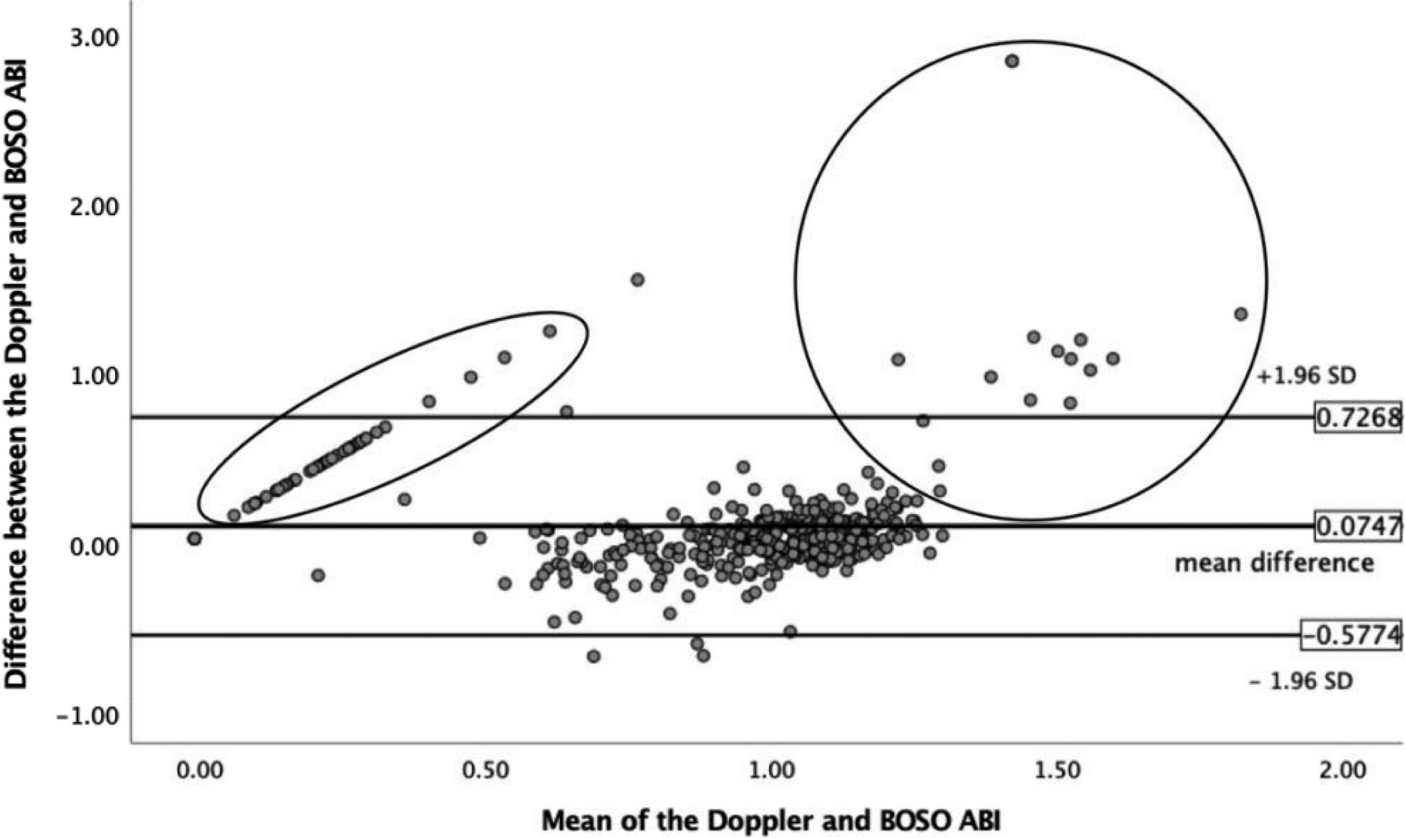
Analysis of the intermodality agreement between the Doppler and BOSO ABI measurements by the Bland–Altman method. The circled part on the left indicates the measurements for which the oscillometric ABI resulted “0,” while the Doppler ABI showed a non-zero value. The circled area on the right demonstrates the cases where Doppler ABI values indicated medial sclerosis, while the BOSO ABI values did not. ABI, ankle-brachial index; SD, standard deviation.

The analysis of the intermodality agreement between the Doppler and BOSO ABI measurements with the Bland–Altman method showed a mean difference of 0.075 between the two methods, with the limits of agreement from −0.577 to 0.727. The linear regression analysis of the differences revealed no proportional bias (*p* = 0.876). Figure 1 highlights the differences with the circled sections resulting from oscillometric “0” readings in which the Doppler ABI values differed from “0,” as well as the cases in which the Doppler ABI values indicated medial sclerosis, while the BOSO ABI values did not. Analyzing these latter 17 limbs, the BOSO ABI was 0.822 ± 0.409. The BOSO ABI recognized five out of the seven PAD limbs at a cutoff level of 0.9 and all seven PAD limbs at a cutoff value of 1.0.

**Figure 2:**
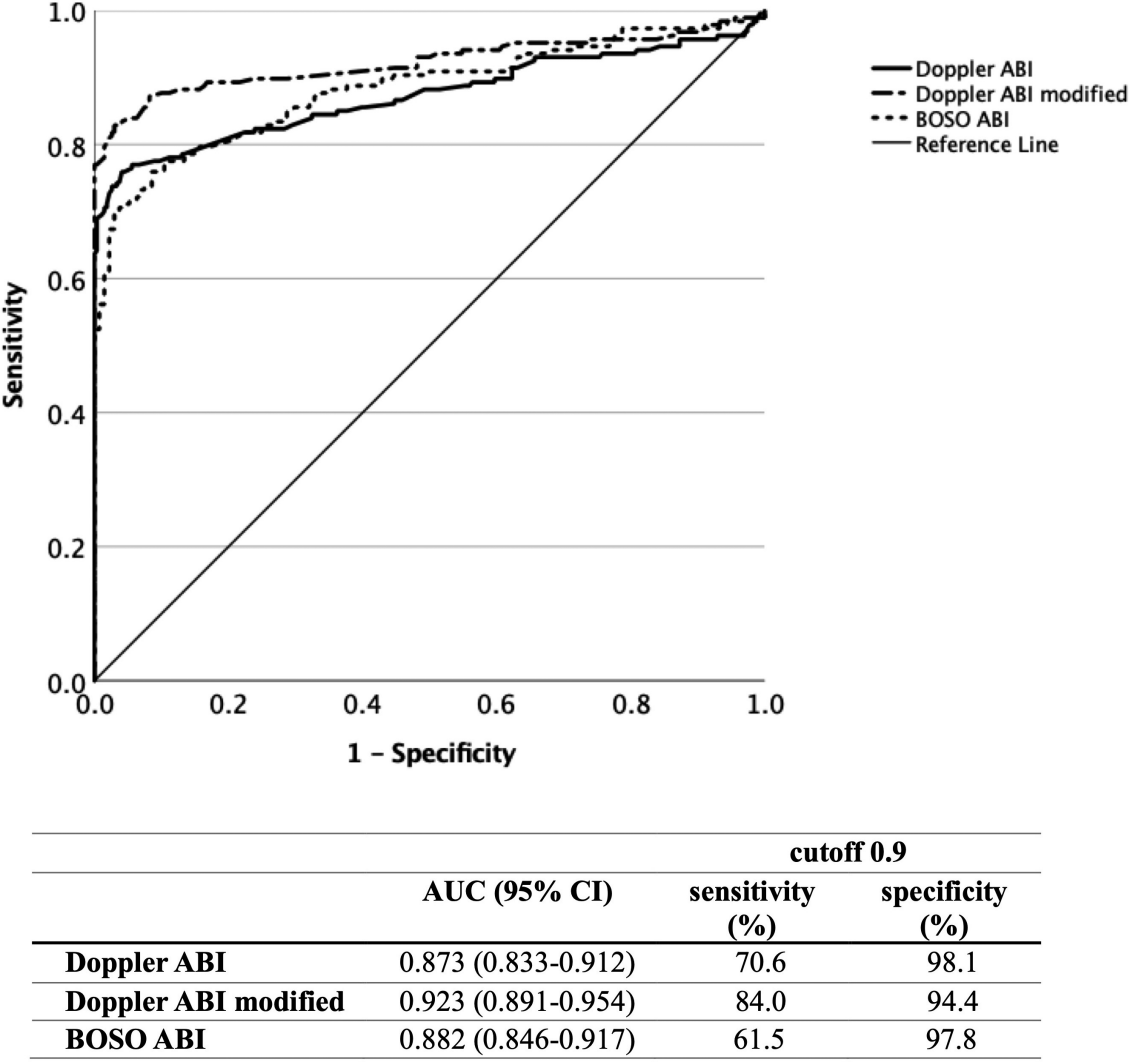
Diagnostic efficacy of the Doppler, modified Doppler, and BOSO ABI measurements by ROC curve analysis with an indication of AUC, sensitivity, and specificity values for all three measurement methods. ABI, ankle-brachial index; ROC, receiver operating characteristic; AUC, area under curve.

The diagnostic efficacy of using the Doppler, modified Doppler, and BOSO ABI values was compared through ROC curve analysis, taking the results of the vascular imaging as a reference (Figure 2). At a cutoff point of 0.9, the Doppler ABI [AUC 0.873 (95% CI 0.833–0.912), *p* < 0.001] showed a sensitivity/specificity of 70.6%/98.1%, the modified Doppler ABI [AUC 0.923 (95% CI 0.891–0.954), *p* < 0.001] showed a sensitivity/specificity of 84.0%/94.4%, and the BOSO ABI [AUC 0.882 (95% CI 0.846–0.917), *p* < 0.001] showed a sensitivity/specificity of 61.5%/97.8%. At a cutoff level of 1.0, the BOSO ABI revealed a sensitivity of 80.7% and a specificity of 79.1%. The optimal cutoff value was considered 0.94 for the Doppler ABI, 0.87 for the modified Doppler ABI, and 0.96 for the BOSO ABI.

Supplementary Figure 1 demonstrates the diagnostic efficacy of the three different ABI measurement methods with an indication of the sensitivity and specificity values at a cutoff level of 0.9 in the high, very high CV risk, and confirmed PAD patient subgroups. No statistically significant differences were found regarding the diagnostic efficacy of the Doppler and modified Doppler ABI assessment between the analyzed subgroups. The AUC values of the BOSO ABI measurement revealed significant differences between high CV risk and confirmed PAD patients (*p* = 0.028) and between very high CV risk and confirmed PAD patients (*p* = 0.041).

**Figure 3:**
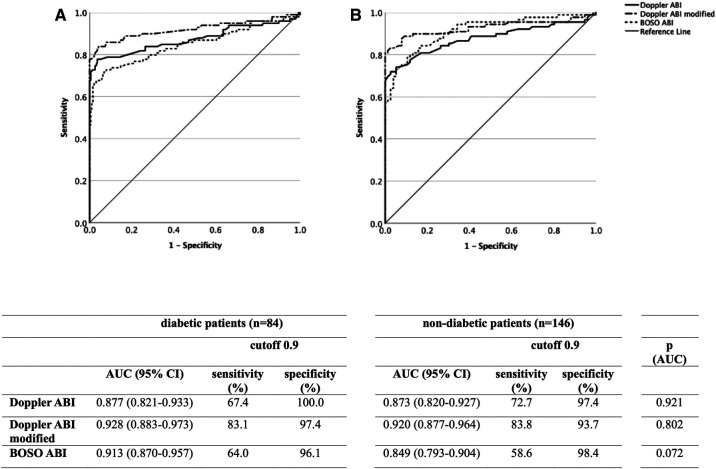
Diagnostic efficacy of the Doppler, modified Doppler, and BOSO ABI measurements in non-diabetic (**A**) and diabetic (**B**) patients by ROC curve analysis with an indication of AUC, sensitivity, and specificity values for all three measurement methods. ABI, ankle-brachial index; ROC, receiver operating characteristic; AUC, area under curve.

Comparing the AUC values of all three methods in diabetic (*n* = 84) and non-diabetic (*n* = 146) patients, no statistically significant differences were found (Figure 3).

### 3.2. ecfPWV measurement

As demonstrated in Supplementary Figure 2, the BOSO device was not able to perform an ecfPWV measurement when the higher BOSO ABI value of the patient's two lower limbs was below 0.9 (*n* = 46). Vascular imaging confirmed atherosclerotic PAD lesions in 100% of these cases. These technically invalid measurements were excluded from the further numerical ROC analysis but were considered clinically important by examining the contribution of the measurement techniques to PAD screening.

**Figure 4:**
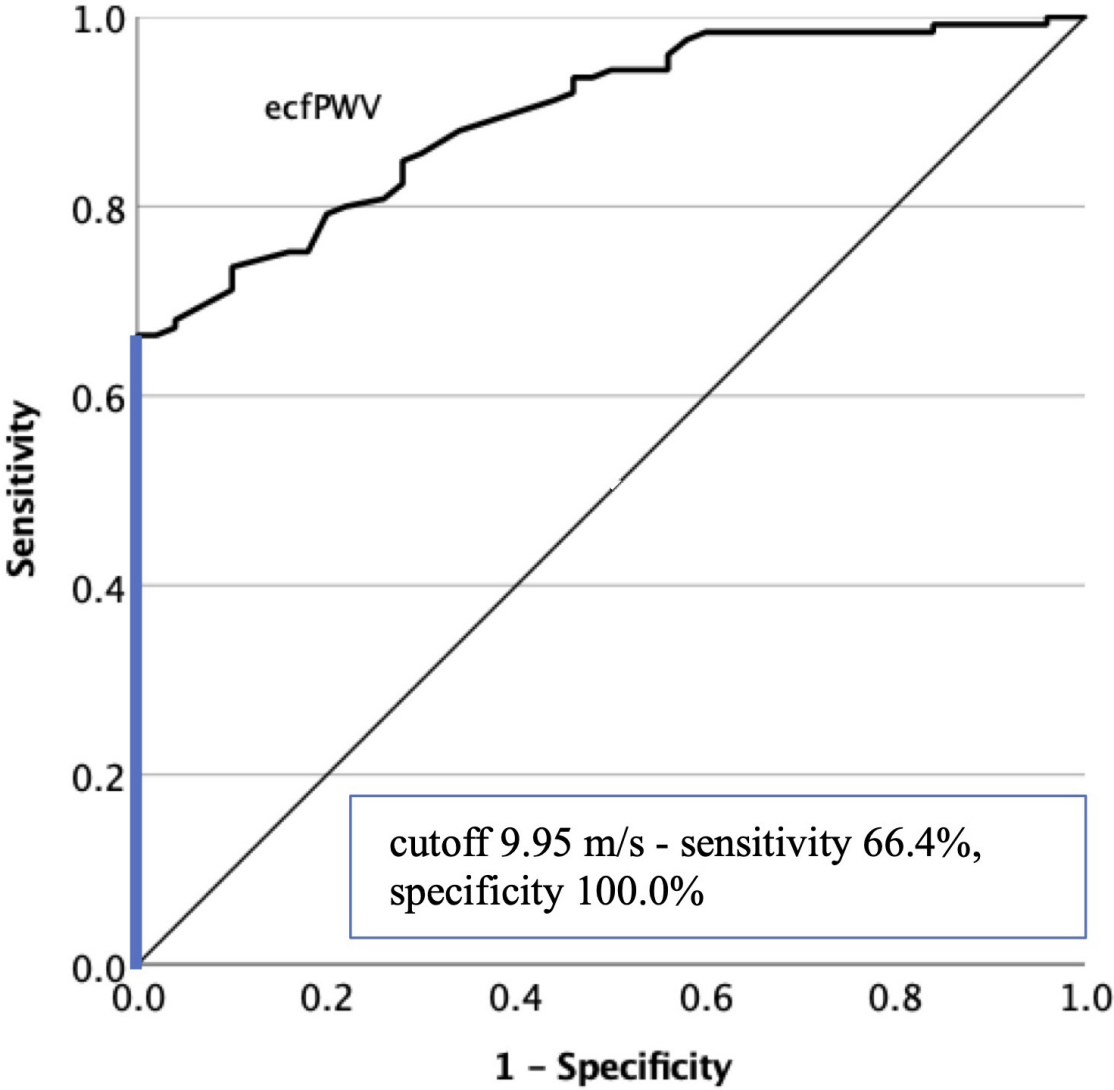
Diagnostic efficacy of the ecfPWV measurement in predicting lower limb atherosclerotic lesions by ROC curve analysis. The optimal cutoff value is indicated on the graph. ecfPWV, estimated carotid-femoral pulse wave velocity; ROC, receiver operating characteristic.

The diagnostic performance of the ecfPWV measurement to predict atherosclerotic lesions was also analyzed with ROC curves (Figure 4). Data analysis with measurable ecfPWV values [AUC 0.896 (95% CI 0.851–0.941), *p* < 0.001] showed that the suggested cutoff level of 10.0 m/s was linked with a sensitivity of 63.2% and a specificity of 100%. The optimal cutoff value of 9.95 m/s practically corresponded to the cutoff value suggested by the manufacturer.

**Figure 5:**
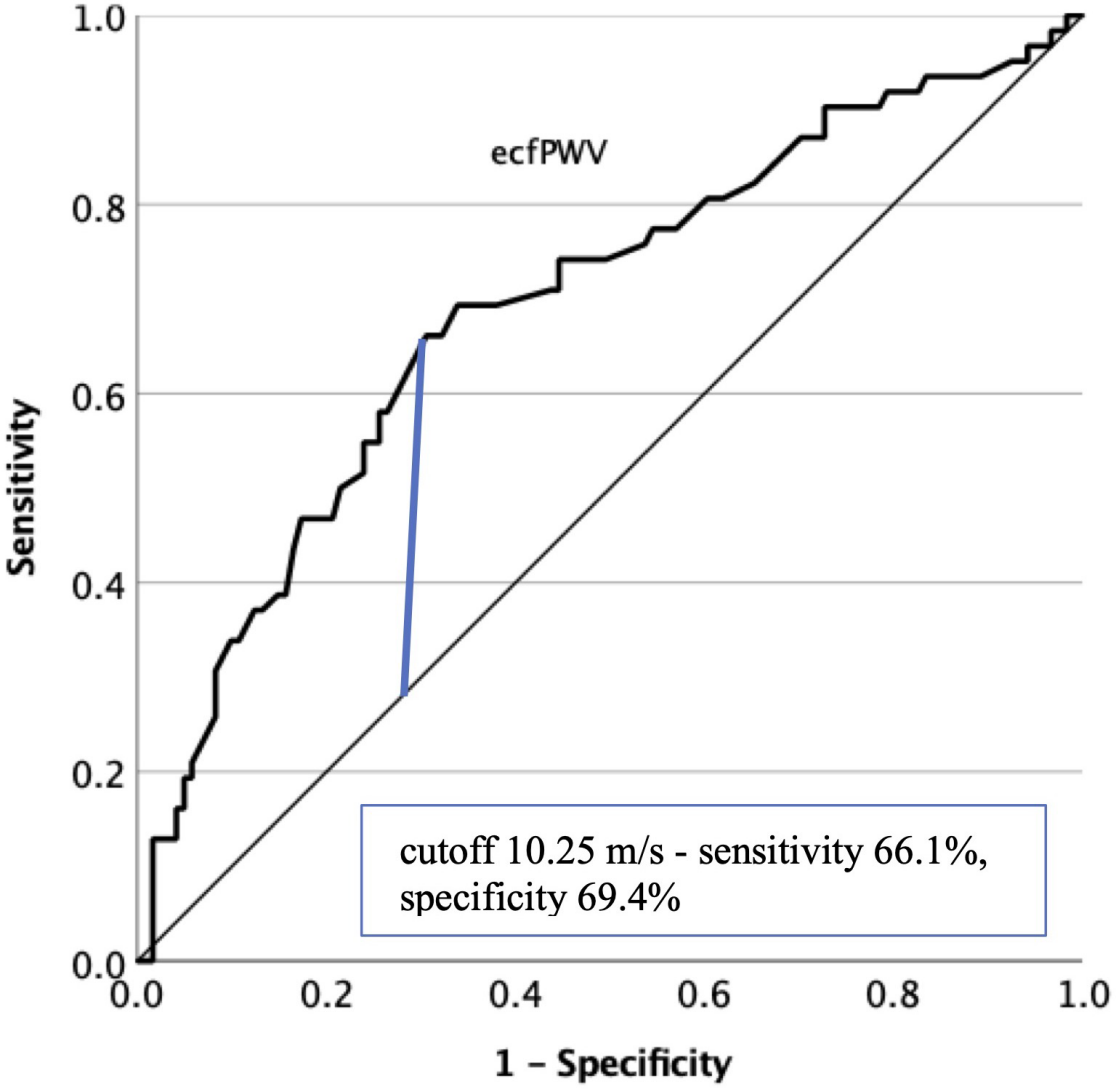
Diagnostic efficacy of the ecfPWV measurement in predicting lower limb PAD with ROC curve analysis. The optimal cut-off value is indicated on the graph. ecfPWV, estimated carotid-femoral pulse wave velocity; PAD, peripheral artery disease; ROC, receiver operating characteristic.

We also aimed to demonstrate whether the ecfPWV measurement contributes to PAD screening. Figure 5 presents the efficiency of measuring ecfPWV in predicting PAD lesions affecting at least one lower extremity [AUC 0.693 (95% CI 0.610–0.776), *p* < 0.001]. At a cutoff level of 10.0 m/s, a sensitivity of 69.4% and a specificity of 66.1% were obtained. The optimal cutoff level was calculated as 10.25 m/s.

### 3.3. Screening with the various methods

Table 2 summarizes the number of patients in the various subgroups screened positive by the Doppler, modified Doppler, and BOSO ABI, LD TBI, and ecfPWV measurements. A cutoff level of 0.9 for ABI measurements and a cutoff level of 0.7 for TBI measurements were used. The last two columns show the results of vascular imaging.

**Table 2:** Patients screened as positive in the different subgroups, compared with the results of vascular imaging.

	Doppler ABI	Doppler ABI modified	BOSO ABI	BOSO ecfPWV	LD TBI	Vascular imaging	
						PAD	Atherosclerosis
Control (*n* = 23)	0	0	0	0	0	0 (0.0%)	2 (8.7%)
Other CV (*n* = 21)	1	1	1	5	8	1 (4.8%)	4 (19.0%)
High CV risk (*n* = 46)	4	5	6	20	22	6 (13.0%)	31 (67.4%)
Very high CV risk (*n* = 65)	17	25	17	40	45	23 (35.4%)	61 (93.8%)

ABI, ankle-brachial index; ecfPWV, estimated carotid-femoral pulse wave velocity; LD, laser Doppler; TBI, toe-brachial index; PAD, peripheral artery disease; CV, cardiovascular.

In the following, we analyzed how the measurement of the ecfPWV contributes to PAD screening. Out of 187 lower extremities affected by PAD (considering both preknown and newly diagnosed cases), Doppler ABI recognized 72.7% and the modified Doppler ABI recognized 84.5%. The discrepancies with the data of the ROC curves result from the ABI values >1.4, which were also considered abnormal. The BOSO ABI was positive in 61.5%, as already shown in the corresponding ROC curve. The ecfPWV measurement gave abnormal results in 82.9% of all PAD patients. If the BOSO ABI was combined with the ecfPWV measurement, 89.5% of the PAD patients were filtered out. If, in addition to the ecfPWV measurement, the cutoff level for the BOSO ABI is raised to 1.0, 92.4% of all PAD patients were recognized by the BOSO device.

TBI measurement proved to be the most effective in PAD screening—with a cutoff level of 0.7, LD TBI was positive in 96.2% and PPG TBI was positive in 94.1% of all PAD limbs.

## 4. Discussion

In our study, we aimed to evaluate the role of an oscillometric, four-limb blood pressure monitor in diagnostics and, in particular, the screening of PAD. We intended to appraise its measurement accuracy compared not only to the standard Doppler but also to the modified Doppler ABI assessment. The sensitivity and specificity of the various methods were analyzed by taking the results of vascular imaging techniques as a reference. We also aimed to analyze the role of the additional ecfPWV measurement in predicting atherosclerotic and PAD lesions, questioning whether it could improve the sensitivity of the tested device in PAD screening.

The measurements of the BOSO ABI-system 100 PWV device are easy to perform, fully automated, and do not require a considerable learning curve. It can point to the potential role of the device in PAD screening, that oscillometric ABI values of the control group differed significantly from values of patients at high or very high CV risk and in patients with confirmed PAD. The ratio of high and very high CV risk patients screened as positive with PAD in our study sample is in good agreement with previous studies (32, 33). Based on our sample, the diagnostic efficacy of all three ABI measurement methods was non-inferior in patients with high or very high CV risk compared to the subgroup of subjects with previously confirmed PAD.

In a 2012 meta-analysis, a significant absolute difference (0.048 ± 0.009) was found between ABI values assessed by the oscillometric vs. Doppler method, which indicated that oscillometric devices measure slightly higher ABI values (12). In contrast to our present study, we found a mean difference of 0.075 ± 0.652 in favor of the Doppler method.

The detailed comparison of the two methods revealed two drawbacks regarding the oscillometric ABI assessment. Since the measurement range of the BOSO device covers 60–240 mmHg, it is unable to detect low ankle pressures, which are reported as “0” mmHg. Although these “0” readings were classified as technically invalid and hindered exact PAD diagnostics, they did not affect the potential role of the BOSO system in screening purposes. Consistent with prior observations, we also found that erroneous oscillometric ABI measurements (results of “0 mmHg”) indicate the presence of a PAD in the affected leg confirmed by vascular imaging (34). The other drawback of the oscillometric measurement compared to the Doppler method (also demonstrated using the Bland–Altman plot) is the failure to detect high ankle pressures, indicating mediasclerosis.

In the literature, limited and partially inconsistent data can be found concerning the agreement between the Doppler and oscillometric ABI measurements using the BOSO ABI-system 100 device. In a study including 50 patients with chronic symptomatic PAD, Diehm et al. analyzed the relationship between the oscillometric, the Doppler, and the modified Doppler ABI methods, respectively, and found a correlation coefficient of 0.77 with the former and 0.75 with the latter; however, patients with incompressible ankle arteries were excluded. The correlation between oscillometric and Doppler ABI values was the highest in non-diabetic patients. Oscillometric readings proved to result in significantly higher ABI values. The time required to perform each test was shown to be significantly longer for the Doppler method (11.4 ± 3.8 min) than for the oscillometric readings (3.9 ± 1.3 min) (24).

Lortz et al. found a highly significant correlation (*r* = 0.904) between BOSO and Doppler ABI values, also in the subgroup of patients with CV diseases (*r* = 0.881) (25). In a study including 839 patients, Wohlfahrt et al. demonstrated a weak agreement (*r* = 0.45 correlation coefficient) between the ABI measurements performed by the BOSO device and the Doppler technique. They also concluded that the oscillometric method overestimated the low and underestimated the high Doppler ABI values (35). The meta-analysis mentioned earlier found a pooled correlation coefficient of 0.71 ± 0.05 between measurements performed by the Doppler method and various oscillometric devices (12). In our present study, a correlation coefficient of 0.614 was found following the correction of the zero measurements.

To determine the sensitivity and specificity of the automatic oscillometric devices, Doppler ABI values were used as a reference in most studies; hence, a sensitivity of 69 ± 6% and a specificity of 96 ± 1% were found (12). In our study, we aimed to provide new acknowledgments by comparing the Doppler, modified Doppler, and oscillometric methods by taking vascular imaging techniques as a reference. Our study also emphasized the importance of determining not only the Doppler ABI but also the modified Doppler ABI, through which a substantially higher sensitivity in PAD diagnostics was achieved.

Thus far, a limited number of other studies are available based on the results of vascular imaging methods. These studies suggested that rather than using the cutoff value of 0.9 generally accepted for the Doppler method, a higher oscillometric ABI cutoff level will prove more appropriate to increase the sensitivity of the oscillometric devices (36–38). We also highlighted that increasing the oscillometric ABI cutoff level from 0.9 to 1.0 would increase the sensitivity from 61.5% to 80.7%, with an acceptable decrease of specificity.

Inconsistent with previous studies (36, 37), none of the ABI measurement methods showed a statistically significant difference between diabetic and non-diabetic patients. The slightly, yet not significantly higher diagnostic efficacy of BOSO ABI may be related to recognizing mediasclerotic PAD limbs with lower ABI values. The ratio of “0 mmHg” measurements was also substantially higher in diabetic patients.

Another important cornerstone of our study was to investigate the role of additional ecfPWV function in screening for atherosclerosis and definitive PAD. Many studies have shown an association between cfPWV and coronary or cerebral atherosclerosis (39); in a meta-analysis, cfPWV was described as an independent predictor of adverse CV events and all-cause mortality (40). However, the association between cfPWV and atherosclerosis of the extremity arteries is less well documented. Moreover, the existing literature presents controversial data regarding the connection between ABI and PWV. Lacroix et al. showed that cfPWV significantly increased with the severity of PAD (41). According to the study of Coutinho et al., higher aortic PWV was associated with lower ABI (42). Catalano et al. also found that PAD patients had higher aortic PWV values than control patients (43). Brewer et al. found that elevated arterial stiffness (aortic augmentation index and pulse pressure) was associated with lower walking distance among claudicants; however, patients with incompressible vessels and severe PAD patients with ABI < 0.5 were excluded (44). Zahner et al. described also significantly higher aortic augmentation index in subjects with PAD (45).

In contrast to these findings, Massmann et al. found that clinically symptomatic PAD was associated with a reduced PWV, which increased following endovascular intervention (46). Brand et al. revealed that changes in PWV in advanced PAD were less well investigated. In severe PAD, indices of the proximal aortic stiffness (aortic augmentation index and central aortic pulse pressure, PPc) may remain increased; however, distal to the arterial stenosis, the distending pressure, and therefore PWV, could also decline. They found that critical limb ischemia was associated with a significantly lower aortic PWV. A mismatch in the PPc/PWV index could indicate an advanced PAD (47). Since we found that the BOSO device was not able to perform an exact ecfPWV measurement in almost half of the confirmed PAD patients, it is not possible to demonstrate any correlation between the ABI and ecfPWV values based on these data. We saw that the device displayed an erroneous ecfPWV measurement when the higher ABI value of the two lower limbs measured by the device was below 0.9. This may support the potential role of the device in PAD screening since 100% of patients with non-measurable ecfPWV were diagnosed with PAD of at least one limb by vascular imaging. However, since the device can only perform the ecfPWV measurement sequential to the measurement of ABI, these patients are practically already screened out based on the ABI measurement. The ROC analysis of the numerically measurable ecfPWV values showed only moderate diagnostic efficacy in predicting PAD lesions. At the same time, ecfPWV proved to be a reliable tool in predicting lower limb atherosclerotic lesions. The cutoff value of 10.0 m/s coincided with the optimal cutoff level established by the ROC analysis and showed an acceptable sensitivity and a specificity of 100% in detecting atherosclerotic plaques, which practically meant that every patient with an ecfPWV greater than or equal to 10.0 m/s was diagnosed with atherosclerotic lesions by the vascular imaging. Therefore, it may contribute to selecting patients at very high CV risk who would benefit from the optimal antiatherosclerosis medical treatment. Canonico et al. highlighted polyvascular artery disease as a common finding in PAD patients; accordingly, half of our PAD patients had atherosclerotic disease at another vascular bed. On the other hand, PAD screening could reveal more multivascular diseases among coronary and cerebrovascular patients who may benefit most from the dual-pathway antithrombotic therapy, besides those who have undergone lower extremity revascularization (48).

By analyzing the contribution of the various methods to PAD screening, detecting TBI by LD or PPG method proved to be the most sensitive. The BOSO ABI measurement alone showed a moderate sensitivity with nearly 100% specificity in detecting at least 50% arterial stenosis of the lower limbs. Using a cutoff level of 1.0 resulted in an increased balanced ratio of sensitivity and specificity (80.7%/79.1%). The automatic ecfPWV measurement may be a promising tool to select patients with lower limb atherosclerosis for further non-invasive PAD testing and optimal medical treatment.

## 5. Study limitations

All measurements of our study were performed by one independent operator. Our study did not aim to test interobserver or intrapatient variability of the various ABI and TBI measurement methods. As a limitation, the use of three different vascular imaging techniques, the subjective evaluation by the color-coded duplex ultrasound examination, and the difficulties in assessing vascular lesions in calf arteries by ultrasound also bear mentioning. The heterogeneity of the “confirmed PAD” group, also including patients with critical limb ischemia or previous amputations, may also limit the study.

## 6. Conclusion

Our study concluded that the tested automatic oscillometric device should not be applied regarding precise PAD diagnostics; however, with the above-mentioned additions, it may significantly contribute to PAD screening by selecting patients who should undergo further non-invasive PAD evaluation. The user-friendly and quick feasibility may make it suitable for PAD screening of a large population.

## Data Availability

The raw data supporting the conclusions of this article will be made available by the authors without undue reservation.

## Footnotes

^1^User instructions boso ABI-system 100 and boso ABI-system 100 PWV. Available at: https://web.archive.org/web/20230720104258/https://www.boso.de/fileadmin/common/pdf/gebrauchsanweisungen/ABI_PWV_4_2.pdf (Accessed July 20, 2023).

^2^SYSTOE, toe systolic blood pressure, finger pressure, TBI. Available at: https://web.archive.org/web/20230718104451/https://www.atysmedical.com/our-products/toe-finger-systolic-pressure-measurement/ (Accessed July 18, 2023).

^3^MedCalc Software Ltd. Comparison of AUC of independent ROC curves. Available at: https://www.medcalc.org/calc/comparison_of_independentROCtest.php (Version 22.013) (Accessed September19, 2023).
