# Supplementary material for: Oscillometric measurement of the ankle-brachial index and the estimated carotid-femoral pulse wave velocity improves the sensitivity of an automated device in screening peripheral artery disease
Source: Front Cardiovasc Med. 2023 Dec 12;10:1275856. doi: 10.3389/fcvm.2023.1275856 (PMC10754531; doi:10.3389/fcvm.2023.1275856)
Supplement: Supplementary file 1 [file Datasheet1.docx]

Supplementary Material

Oscillometric measurement of the ankle-brachial index and the estimated carotid-femoral pulse wave velocity improves the sensitivity of an automated device in screening peripheral artery disease

Krisztina Fendrik^*^, Katalin Biró, Dóra Endrei, Katalin Koltai, Barbara Sándor, Kálmán Tóth, Gábor Késmárky

*** Correspondence:** fendrik.krisztina@pte.hu

# Supplementary Figures and Tables

## Supplementary Figures


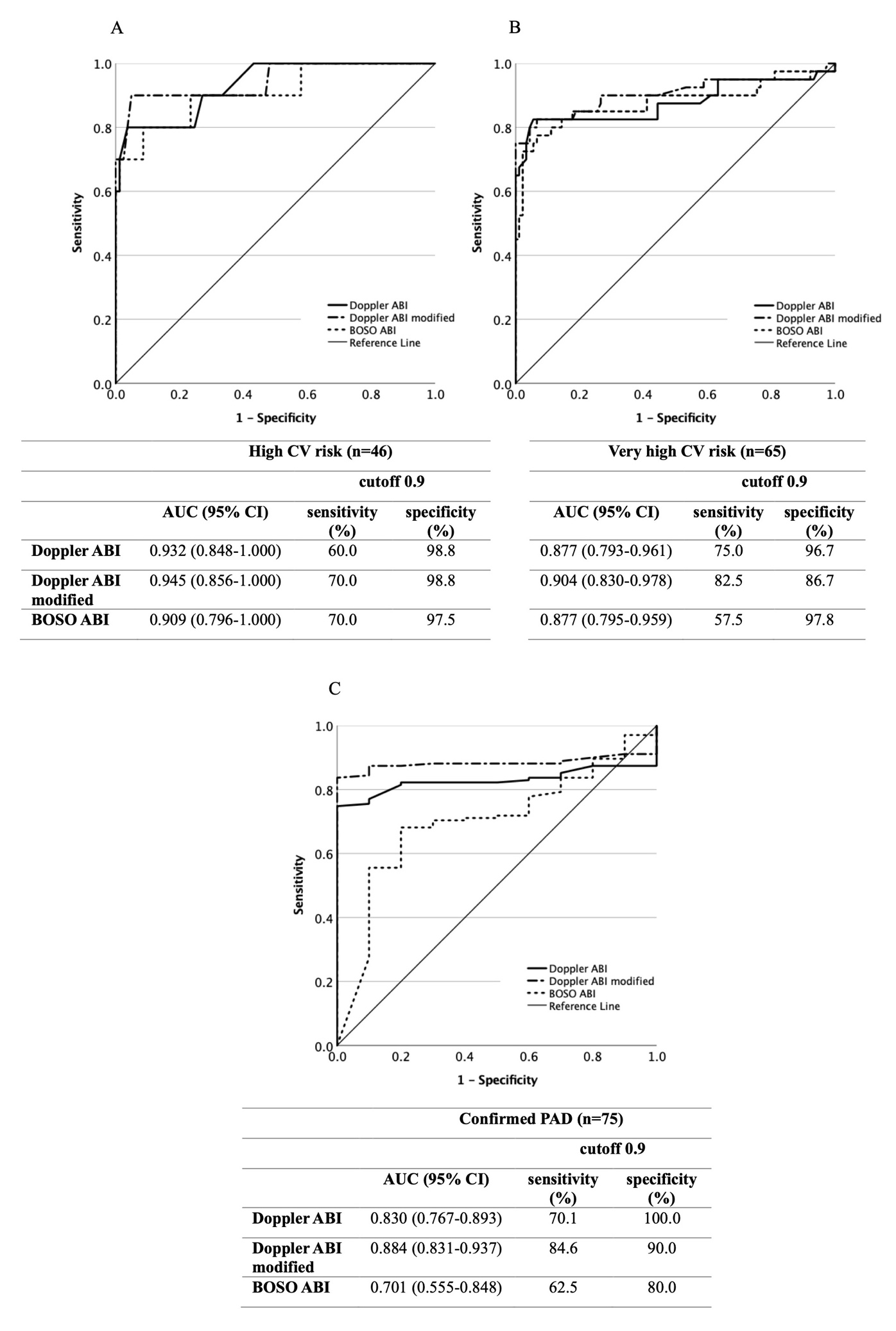


**Supplementary Figure 1.** Diagnostic efficacy of the Doppler, modified Doppler and BOSO ABI measurements in the subgroups of patients with high CV risk (A), with very high CV risk (B) and in patients with previously confirmed PAD (C) by ROC curve analysis with an indication of AUC, sensitivity and specificity values ​​for all three measurement methods (ABI, ankle-brachial index; CV, cardiovascular; ROC, receiver operating characteristic; AUC, area under curve; PAD, peripheral artery disease).

**
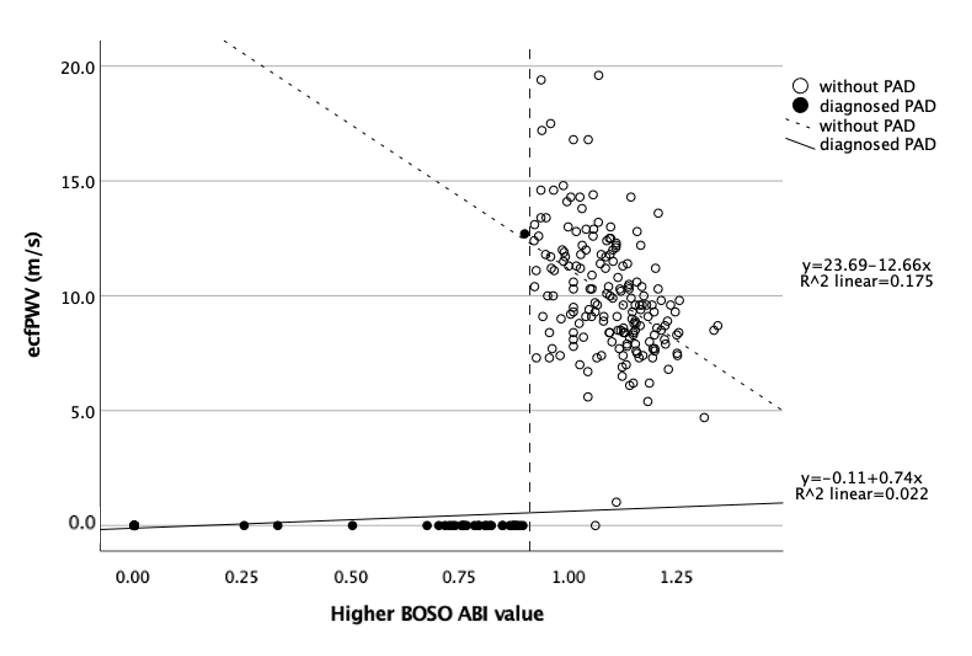
**

**Supplementary Figure 2.** The connection between the higher ABI value of the two lower limbs and the ecfPWV measurement performed by the BOSO device - if the higher BOSO ABI value of the patient’s two lower limbs was below 0.9, the ecfPWV measurement did not result in numerical data. (ABI, ankle-brachial index; PAD, peripheral artery disease; ecfPWV, estimated carotid-femoral pulse wave velocity)

## Supplementary Tables

**Table 1** Demographics and baseline characteristics of the study population. “p” values refer to the comparison of five subgroups (SD, standard deviation; BMI, body mass index; DOAC, direct oral anticoagulant; ACEI, angiotensin-converting enzyme inhibitor; ARB, angiotensin receptor blocker; CV, cardiovascular).

|  |  | Control | Other CV | High CV risk | Very high CV risk | Confirmed PAD | All patients | p |
| --- | --- | --- | --- | --- | --- | --- | --- | --- |
|  |  | (n=23) | (n=21) | (n=46) | (n=65) | (n=75) | (n=230) |  |
| Age (mean ± SD years) | | 59.5 ± 11.9 | 44 ± 15.3 | 62.0 ± 10.1 | 70.3 ± 8.5 | 67.3 ± 13.4 | 64.1 ± 13.7 | <0.001 |
| Male sex (No. %) | | 10 (43.5%) | 8 (38.1%) | 17 (37.0%) | 28 (43.1%) | 38 (50.7%) | 102 (44.3%) | 0.627 |
| BMI (mean ± SD kg/m2) | | 25.8 ± 4.8 | 29.0 ± 9.3 | 31.6 ± 7.6 | 29.3 ± 10.3 | 27.3 ± 5.6 | 28.5 ± 6.7 | 0.003 |
|  | | | |  |  |  |  |  |
| Co-morbidities and risk factors (No. %) | | | |  |  |  |  |  |
| Hypertension |  | 9 (39.1%) | 5 (23.8%) | 40 (87.0%) | 62 (95.4%) | 65 (86.7%) |  | <0.001 |
| Diabetes mellitus | | 0 (0%) | 0 (0%) | 25 (54.3%) | 27 (41.5%) | 32 (42.7%) |  | <0.001 |
| Diabetic polyneuropathy | | 0 (0%) | 0 (0%) | 5 (10.9%) | 8 (12.3%) | 10 (13.3%) |  | 0.182 |
| Dyslipidemia | | 3 (13.0%) | 2 (9.5%) | 28 (60.9%) | 53 (81.5%) | 63 (84.0%) |  | <0.001 |
| Smoker (current) | | 0 (0%) | 4 (19.0%) | 11 (23.9%) | 18 (27.7%) | 19 (25.3%) |  | 0.085 |
| Smoker (former) | | 4 (17.4%) | 2 (9.5%) | 11 (23.9%) | 19 (29.2%) | 35 (46.7%) |  | 0.003 |
| Coronary heart disease | | 0 (0%) | 0 (0%) | 0 (0%) | 31 (47.7%) | 22 (29.3%) |  | <0.001 |
| Carotid artery disease | | 0 (0%) | 0 (0%) | 0 (0%) | 19 (29.2%) | 16 (21.3%) |  | <0.001 |
| Cerebrovascular events | | 0 (0%) | 0 (0%) | 0 (0%) | 9 (13.8%) | 7 (9.3%) |  | 0.016 |
| Renal artery disease | | 0 (0%) | 0 (0%) | 0 (0%) | 1 (1.5%) | 3 (4.0%) |  | 0.426 |
| Abdominal aortic disease | | 0 (0%) | 0 (0%) | 0 (0%) | 1 (1.5%) | 1 (1.3%) |  | 0.859 |
|  |  |  |  |  |  |  |  |  |
| Concomitant medication (No. %) | | |  |  |  |  |  |  |
| Aspirin |  | 1 (4.3%) | 4 (19.0%) | 10 (21.7%) | 20 (30.8%) | 34 (45.3%) |  | 0.001 |
| Clopidogrel |  | 0 (0%) | 0 (0%) | 1 (2.2%) | 20 (30.8%) | 43 (57.3%) |  | <0.001 |
| Cilostazol |  | 0 (0%) | 0 (0%) | 0 (0%) | 0 (0%) | 16 (21.3%) |  | <0.001 |
| Oral anticoagulants | |  |  |  |  |  |  |  |
| Vitamin K antagonist | | 0 (0%) | 1 (4.8%) | 1 (2.2%) | 3 (4.6%) | 1 (1.3%) |  | 0.666 |
| DOAC |  | 0 (0%) | 5 (23.8%) | 16 (34.8%) | 25 (38.5%) | 15 (20.0%) |  | 0.003 |
| ACEI/ARB |  | 9 (39.1%) | 4 (19.0%) | 36 (78.3%) | 48 (73.8%) | 52 (69.3%) |  | <0.001 |
| Beta blocker | | 3 (13.0%) | 6 (28.6%) | 19 (41.3%) | 43 (66.2%) | 47 (62.7%) |  | <0.001 |
| Calcium channel blocker | | 3 (13.0%) | 4 (19.0%) | 21 (45.7%) | 25 (38.5%) | 38 (50.7%) |  | 0.005 |
| Statin |  | 3 (14.3%) | 2 (9.5%) | 17 (37.0%) | 44 (67.7%) | 58 (77.3%) |  | <0.001 |
| Fibrate |  | 0 (0%) | 0 (0%) | 4 (8.7%) | 1 (1.5%) | 1 (1.3%) |  | 0.07 |
| Ezetimibe |  | 0 (0%) | 0 (0%) | 1 (2.2%) | 7 (10.8%) | 2 (2.6%) |  | 0.05 |
|  |  |  |  |  |  |  |  |  |
| Lower limb symptoms (No. %) | | |  |  |  |  |  |  |
| Leg ulcers |  | 0 (0%) | 0 (0%) | 1 (2.2%) | 1 (1.5%) | 13 (17.3%) |  | <0.001 |
| Ischemic rest pain | | 0 (0%) | 0 (0%) | 0 (0%) | 3 (4.6%) | 15 (20.0%) |  | <0.001 |
| Intermittent claudication | | 0 (0%) | 1 (4.8%) | 3 (6.5%) | 14 (21.5%) | 39 (52.0%) |  | 0.015 |
| PAD history (No. %) | | |  |  |  |  |  |  |
| Percutaneous transluminal angioplasty |  | 0 (0%) | 0 (0%) | 0 (0%) | 0 (0%) | 32 (42.7%) |  | <0.001 |
| Bypass surgery | | 0 (0%) | 0 (0%) | 0 (0%) | 0 (0%) | 11 (14.7%) |  | <0.001 |
| Other vascular surgery | | 0 (0%) | 0 (0%) | 0 (0%) | 0 (0%) | 11 (14.7%) |  | <0.001 |
| Major amputation | | 0 (0%) | 0 (0%) | 0 (0%) | 0 (0%) | 4 (5.3%) |  | 0.078 |
| Minor amputation | | 0 (0%) | 0 (0%) | 0 (0%) | 0 (0%) | 7 (9.3%) |  | 0.005 |
